# Modeling of corona virus and its application in confocal microscopy

**DOI:** 10.1186/s43088-022-00276-2

**Published:** 2022-08-13

**Authors:** Abdallah Mohamed Hamed

**Affiliations:** Phys Deptartment, Fac. Sci., Ain Shams University, Cairo, 11566 Egypt

**Keywords:** Corona virus, Confocal laser microscope, Spiky circular apertures, Impulse response

## Abstract

**Background:**

The proposal of spiky apertures showed resolution improvement compared with the circular apertures. Three models of corona virus are given. The 1st model consists of uniform circular aperture provided with 8 spikes while the 2nd model has 16 spikes for the same uniform circular aperture. The 3rd model has circular linear distribution with 8 spikes.

**Results:**

The Normalized Point Spread Function (PSF) or the impulse response is computed for the three models using fast Fourier transform technique. In addition, the autocorrelation function corresponding to these apertures is calculated and compared with that corresponding to the ordinary circular and conic apertures. Coronavirus image is used as an object in the formation of images using confocal scanning laser microscope provided with suggested models. The fabricated MATLAB code is used to compute and plot all images and line plots.

**Conclusions:**

The PSF plots are computed from Eqs. (8) and (12) using MATLAB code showing narrower cutoff in the PSF for spiky aperture compared with that corresponding to the uniform circular aperture and modulated linear and quadratic apertures. Hence, I reached resolution improvement in the case of spiky aperture.

## Background

There is resolution improvement by using annular aperture as compared with the open circular aperture shown [1]. In addition, we applied aperture modulation using linear, quadratic, and other types of amplitude modification inside the circular aperture to a confocal laser microscope [2–7] for further resolution improvement in the microscope. Optimization of axial resolution in confocal imaging using annular pupils [8] is performed.

The principle of aperture modulation and intensity extrapolation (AMIE) are described in a recent report [9] as a sequence of images obtained with the same imaging system but different aperture sizes. The image sequence is used to fit the intensity function of the aperture size at each position on the image plane. Then, there is fitted intensity function extrapolation to the aperture size larger than the maximum one of the imaging systems. Finally, a super-resolution (SR) image [10–14] is reconstructed with the extrapolated intensity values of all position on the image plane. The critical point of the method is how to achieve the fitting process of the intensity function with the image sequence, and the key assumption is that the intensity function is analytic and continuous.

Different approaches for predicting the spread of COVID-19 epidemic in different countries outlined in recent publication [15–19] are summarized in the next paragraph.

A mathematical model investigates the impact of unreported cases of the COVID-19 in three North African countries: Algeria, Egypt, and Morocco [15]. In this issue, massive infection in the population is created if the control measures are not respected (such as wearing the face mask and social distancing), while the progress of the COVID-19 epidemic is reported in the modeling in the USA, UAE, and Algeria [16]. The main propagation of the COVID-19 to find the control for the rapid spread of this viral disease in real life a discrete form of the model considers the dynamics of population in four classes [17]. A robust study of a piecewise fractional-order COVID-19 mathematical model with quarantine class and vaccination using SEIQR epidemic model [18] is performed. The evolution of the COVID-19 infection cases is predicted the spread of COVID-19 disease in Algeria and India using the least square method [19].

Meanwhile, my work deals with the image processing of corona virus using modulated shapes as the COVID-19. The interest of my work uses spiky aperture as the COVID- 19 shape applying it in the formation of images using confocal scanning laser microscope (CSLM). Hence, we proposed spiky models of apertures as the Omicron COVID19 images [20] used in the formation of images in (CSLM) [6, 7]. The method is followed by results and discussions, and finally a conclusion.

## Methods

**Fig. 1:**
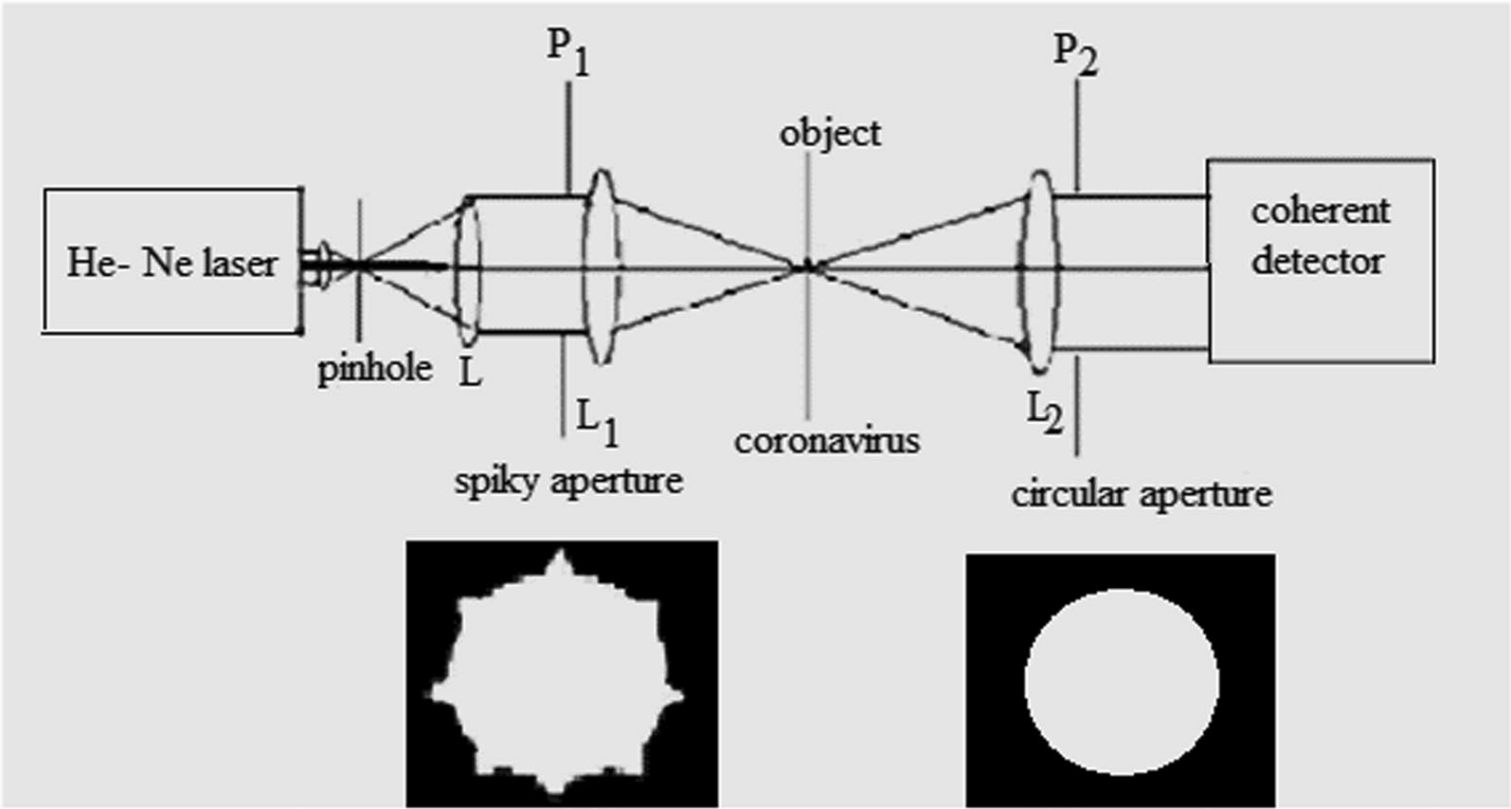
Set-up for the confocal scanning laser microscope provided with illumination spiky aperture P_1_ and detection uniform circular aperture P_2_. The He-Ne laser made parallel beam using spatial filter composed with adequate pinhole and collimating lens L. The object is mechanically scanned with the electronic scanning of the detector to construct the image

A collimated beam from He-Ne laser is obtained using spatially filtered techniques as shown in Fig. 1. The collimated parallel beam is incident on the confocal arrangement of the microscope [2, 3, 6, 7]. In this confocal arrangement, the mechanical scanning synchronized with the electronic scanning in the detection plane allows to construct the image. The formation of images using scanned object (omicron corona virus) is summarized as follows:

Consider unit amplitude of coherent radiation incident upon the 1st spiky aperture P_1_ (u, v) composed of a circle surrounded by irregular spikes. Then, the transmitted complex amplitude is represented as follows:

1 $$\begin{aligned} {\text{P}}_{1} \left( {{\text{u}},v} \right) & = & 1{ };{\text{ for }}\frac{{\rho}}{\rho{_{{0{ }}} + \epsilon_{r} }} \le 1;{ \epsilon_{r}<< \rho{_{{0{ }}} }} \\ = {\text{ zero}};{\text{ otherwise}} \\ \end{aligned}$$

ρ: is the radial coordinate in the aperture plane,${\uprho }_{0}$: the internal aperture radius surrounded by the spikes of average width $_{r}$. Hence, the external radius is ${\uprho }_{{0{ }}} +\varepsilon_{r}$ and the ratio between the internal and external radii is $\alpha= \frac{{\uprho }_{0}}{{\left({\uprho }_{{0{ }}} +\varepsilon_{r} \right)}}$.

In the front focal plane of the converging lens L_1_, we get by applying the Fourier transform (F.T.) upon Eq. (1) the following:

2 $${\text{h}}_{1} \left( {{\text{x, y}}} \right) = {\text{F.T.}} \left\{ {{\text{P}}_{1} \left( {{\text{u, v}}} \right)} \right\} = \mathop \smallint \limits_{ -\infty\ }^{{\infty}} \mathop \smallint \limits_{ -\infty }^{{\infty}} {\text{P}}_{1} \left( {{\text{u, v}}} \right) \exp \left[ { - \frac{j2\pi}{f\lambda}\left( {ux + vy} \right)} \right] du dv$$

where ${\text{h}}_{1} \left( {{\text{x}},{\text{y}}} \right)$ is the PSF or amplitude impulse response corresponding to the irregular aperture of random spikes P_1_.

The F.T. in Eq. (2), for the irregular spiky aperture, is rewritten in integral radial form as follows:

3 $${\text{h}}_{1} \left( {{\text{x, y}}} \right) = \mathop \smallint \limits_{0}^{2\pi}\ \mathop \smallint \limits_{0}^{{\infty}} {\text{P}}_{1} \left( {{\text{u, v}}} \right)\exp \left[ { - j\left( \frac{2\pi}{f\lambda} \right){{\rho}\text{r}}\cos \left(\theta -\phi \right)} \right] \rho{d\rho} {\text{ d}\theta}$$

Substitute from Eq. (1) in Eq. (3), we get:

4 $${\text{h}}_{1} \left( {{\text{x}},{\text{y}}} \right) = \mathop \smallint \limits_{0}^{2\pi} \mathop \smallint \limits_{0}^{{\rho_{{0{ }}} + \epsilon_{r} }} \exp \left[ { - j\left( \frac{2\pi}{f\lambda} \right){ \rho}{\text{ r}}\cos \left(\theta-\phi\right)}\right]\rho{d\rho}{\text{ d}\theta}$$

where $x = r\cos\phi$, $y = r\sin\phi$ , and $r = \sqrt {x^{2} + y^{2} }$. The polar coordinates in the aperture plane (*ρ*, *θ*) are related to the cartesian coordinates (u, v) as follows:

$${\text{u}} = \rho\cos {\theta },$$ $${\text{v}} = \rho\sin {\theta },{ }$$ and $$\rho= \sqrt {u^{2} + v^{2} }$$.

Assuming spherical symmetry of revolution for the aperture and hence using Bessel identity Eq. (4) becomes:

5 $${\text{h}}_{1} \left( {{\text{x}},{\text{y}}} \right) = 2\pi \mathop \smallint \limits_{0}^{{\rho_{0} + { }\epsilon_{r} }} { }J_{0} \left( {\frac{{2\pi\rho{\text{r}}}}{{\text{f}\lambda}}} \right) \rho{\text{ d}\rho}$$

where *J*_0:_ Bessel function of zero order.

The above integral is solved by separation into two parts as follows:

6 $${\text{h}}_{1} \left( {{\text{x}},{\text{y}}} \right) = 2\pi \mathop \smallint \limits_{0}^{{\rho_{0} }} { }J_{0} \left( {\frac{{2\pi{\rho}{\text{r}}}}{{\text{f}\lambda}}} \right) {\rho }{\text{ d }\rho} + { }2\pi \mathop \smallint \limits_{{\rho_{0} }}^{{\rho_{0} + { }\epsilon_{r} }} { }J_{0} \left( {\frac{{2\pi{\rho }{\text{r}}}}{{\text{f}\lambda}}} \right) {\rho }{\text{ d }\rho}$$

The solution of the above integral from zero to $\rho_{0}$ gives the known Airy disc, and we get the following:

7 $${\text{h}}_{1} \left( {{\text{x}},{\text{y}}} \right) = \frac{{2J_{1} \left( w \right)}}{w} + { }2\pi \mathop \smallint \limits_{{\rho_{0} }}^{{\rho_{0} + { }\epsilon_{r} }} { }J_{0} \left( {\frac{{2\pi{\rho }{\text{r}}}}{{\text{f}\lambda}}} \right) {\rho }{\text{ d }\rho}$$

where *w* is the reduced coordinate given by $w = \frac{{2{\pi }\rho_{{0{ }}} {\text{r}}}}{{\text{f}\lambda}}$.

The 2nd integral is approximated by annulus of random surface; hence, we finally get for the PSF the following formula:

8 $${\text{h}}_{1} \left( {\text{w}} \right) = \frac{{2J_{1} \left( w \right)}}{w} + {\beta }J_{0} \left( w \right); \beta< 1$$

It is assumed that for the spiky pattern it is approximated by annular distribution where the value of the parameter $= 0.5$.

Accurate solution obtained for the second integral corresponding to the spiky part is computed from the difference between the computed values corresponding to the external and internal radii. Hence, from Eq. (7), we get the following result for the PSF [21].

9 $${\text{h}}_{1} \left( {\text{w}} \right) = \frac{{2J_{1} \left( w \right)}}{w} + \frac{2}{{1 -\alpha^{2} }} \left[ {\frac{{J_{1} \left( {w_{1} } \right)}}{{w_{1} }} -\alpha \frac{{J_{1} \left( w \right)}}{w}} \right]$$

The reduced coordinate w_1_ is related to the reduced coordinate w by the formula:

10 $$w_{1} = \frac{{2{\pi }(\rho_{{0{ }}}+ { \epsilon}_{r} ){\text{r}}}}{{\lambda}\text{f}}= w(1+\frac{{\epsilon}_{r}}{\rho_{{0 }}})$$

The omicron corona virus image is placed in the object plane of transmitted amplitude $\mathrm{g}(\mathrm{x},\mathrm{y})$.

The 2nd lens shown conjugate to the 1^st^ lens where the pupil aperture ${P}_{2}\left(u,v\right)$ are placed in front of the 2nd lens. The point spread function is equal ${\mathrm{h}}_{2}\left(\mathrm{x},\mathrm{y}\right)$ for uniform circular aperture represented as:

11 $$\begin{aligned} {\text{P}}_{2} \left( {{\text{u}},{\text{ v}}} \right) & = 1{ };{\text{ for }}\mid\frac{{\rho}}{\rho{_{{0{ }}} }}\mid \le 1 \\ = {\text{ zero}};{\text{ otherwise}} \\ \end{aligned}$$

Hence, operating F.T. upon Eq. (11), we get the known result of Airy disc represented as follows:

12 $${\text{h}}_{2} \left( {\text{w}} \right) = \frac{{2J_{1} \left( w \right)}}{w}$$

where *w* is the reduced coordinate as given in Eq. (7).

The object ${\text{g}}\left( {{\text{x}},{\text{y}}} \right)$ is placed in the common short focus corresponding to the two microscope lenses. Consequently, the scanned object transparency g (x, y) is convoluted by both the PSF’s. Hence, we get the following convolution in the object plane of transmitted complex amplitude C (x, y) written in integral form as follows:

13 $${\text{C}}\left( {{\text{x}},{\text{y}}} \right) = { }\mathop \smallint \limits_{ - }^{{}} \mathop \smallint \limits_{ - }^{{}} {\text{g}}\left( {{\text{x}}_{s} , {\text{y}}_{s} } \right) {\text{h}}_{1} \left( {{\text{x}} - {\text{x}}_{s} { },{\text{y}} - {\text{ y}}_{s} { }} \right).{\text{h}}_{2} \left( {{\text{x}} - {\text{ x}}_{s} ,{\text{y}} - {\text{ y}}_{s} } \right) {\text{dx}}_{s} {\text{dy}}_{s}$$

The mechanical scanning in the object plane is represented by ($$x_{s} ,y_{s} ).$$

Equation (13) is written symbolically as follows:

14 $${\text{C}}\left( {{\text{x}},{\text{y}}} \right) = { }\{ {\text{h}}_{1} \left( {{\text{x}},{\text{y}}} \right).{\text{h}}_{2} \left( {{\text{x}},{\text{y}}} \right)\} {\text{ g}}\left( {{\text{x}},{\text{y}}} \right)$$

where $$symbol for convolution product.$$

The multiplication of

15 $${\text{ h}}_{1} \left( {{\text{x}},{\text{y}}} \right).{\text{h}}_{2} \left( {{\text{x}},{\text{y}}} \right) = {\text{h}}_{{{\text{eff}}}} \left( {{\text{x}},{\text{y}}} \right)$$

Substitute from Eqs. (8, 12), we get the following resultant PSF:

16 $${\text{h}}_{{{\text{eff}}}} \left( {\text{w}} \right) = \left[ {\frac{{2J_{1} \left( w \right)}}{w}} \right]^{2} +\beta J_{0} \left( {\text{w}} \right) \frac{{2J_{1} \left( w \right)}}{w}$$

${\text{h}}_{{{\text{eff}}}} \left( {{\text{x}},{\text{y}}} \right)$ is the effective or resultant PSF in confocal microscope using the illuminating aperture P_1_ of irregular spiky shape while the 2nd aperture corresponding the detector has uniform circular shape. It is shown that the 2nd term in the R.H.S. of Eq. (16) is the contribution of the spiky part of the aperture upon the ${\text{h}}_{{{\text{eff}}}} \left( {\text{w}} \right).$

The detected intensity is represented as the modulus square of the complex amplitude $C\left( {x,y} \right),$ represented as follows:

17 $${\text{I}}\left( {{\text{x}},{\text{y}}} \right) = \mid{\text{ C}}\left( {{\text{x}},{\text{y}}} \right){ }\mid^{2} = \mid{\text{ h}}_{{{\text{eff}}}} \left( {{\text{x}},{\text{y}}}\right){\text{ g}}\left( {{\text{x}},{\text{y}}} \right){ }\mid^{2}$$

Equation (17) is the intensity distribution in case of Confocal Scanning Laser Microscope (CSLM).

## Results

Setup for the confocal scanning laser microscope is provided with illumination spiky aperture P_1_ and detection uniform circular aperture P_2_. The He-Ne laser made parallel beam using spatial filter composed with adequate pinhole and collimating lens L. The object mechanically is scanned with the electronic scanning of the detector to construct the image as shown in Fig. 1.

**Fig. 2:**
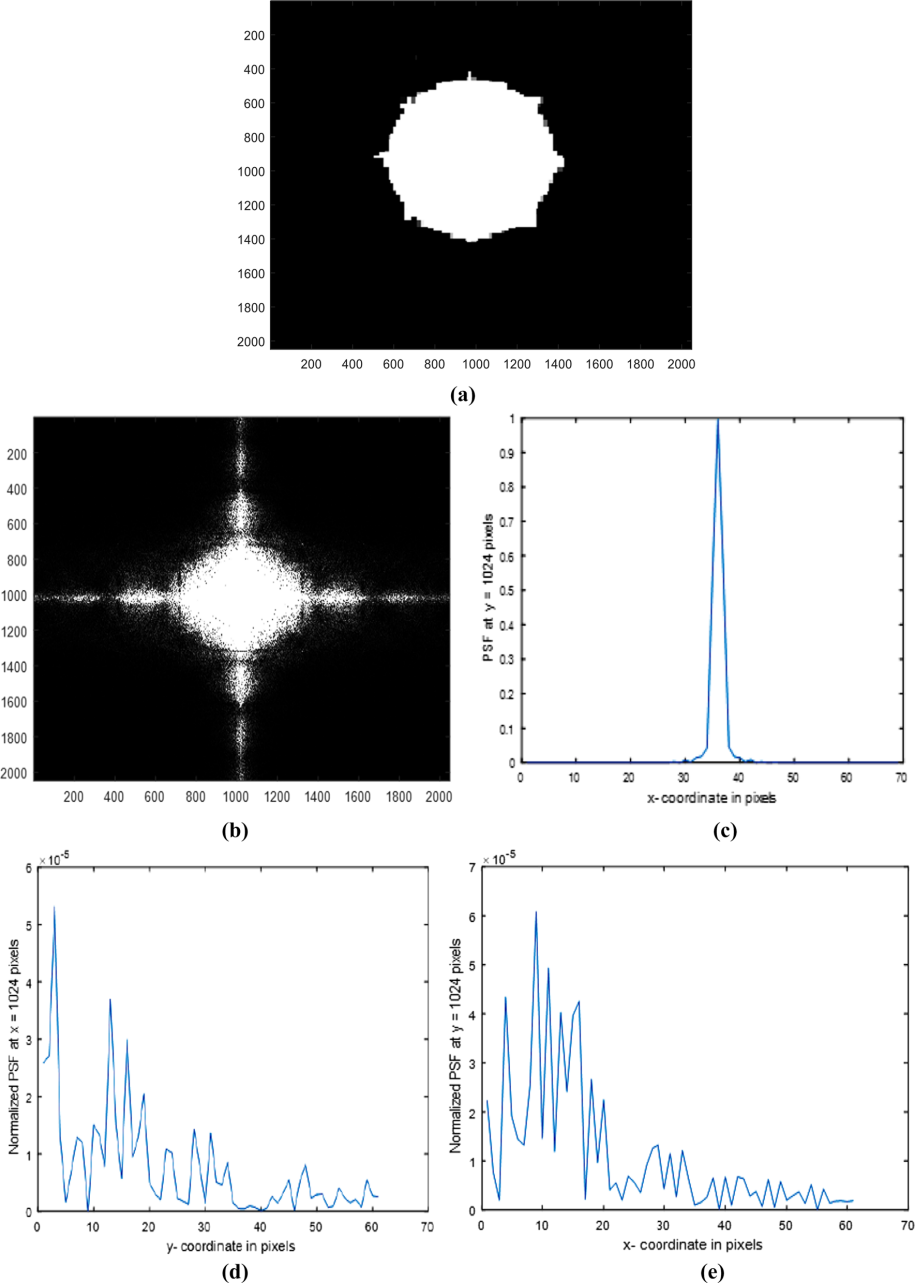
**a**: Circular aperture with irregular spikes in a matrix of dimensions 2048 × 2048 pixels. The average diameter = 900 pixels. **b**: The diffracted image or the intensity impulse response corresponding to the aperture is shown in (**a**). **c**: The Normalized PSF plot in the range from 990 up to 1058 pixels obtained from the image is shown in the (**b**). **d**: The PSF, for the 1st model in the range from 1040 up to 1100 pixels. The profile is plotted at constant *x* = 1024 pixels. **e**: The PSF, for the 1st model in the range from 1040 up to 1100 pixels. The profile is plotted at constant *y* = 1024 pixels

Irregular circular aperture with 8 spikes in a matrix of dimensions 2048 × 2048 pixels is fabricated and plotted as shown in Fig. 2a. The average diameter = 900 pixels. The diffracted image or the intensity impulse response corresponding to the aperture shown in Fig. 2a is computed using FFT and plotted as shown in Fig. 2b. The Normalized PSF plot in the range from 990 up to 1058 pixels is obtained from the image shown in Fig. 2b and plotted as shown in Fig. 2c. It is shown that the central peak is found at 36 pixels. The PSF for the 1st model in the range from 1040 up to 1100 pixels outside the center found at 1024 pixels is obtained from the diffracted image and plotted as shown in Fig. 2d. The profile is plotted at constant *x* = 1024 pixels, while the PSF at constant *y* = 1024 pixels from 1040 up to 1100 pixels outside the center is plotted as shown in Fig. 2e.

**Fig. 3:**
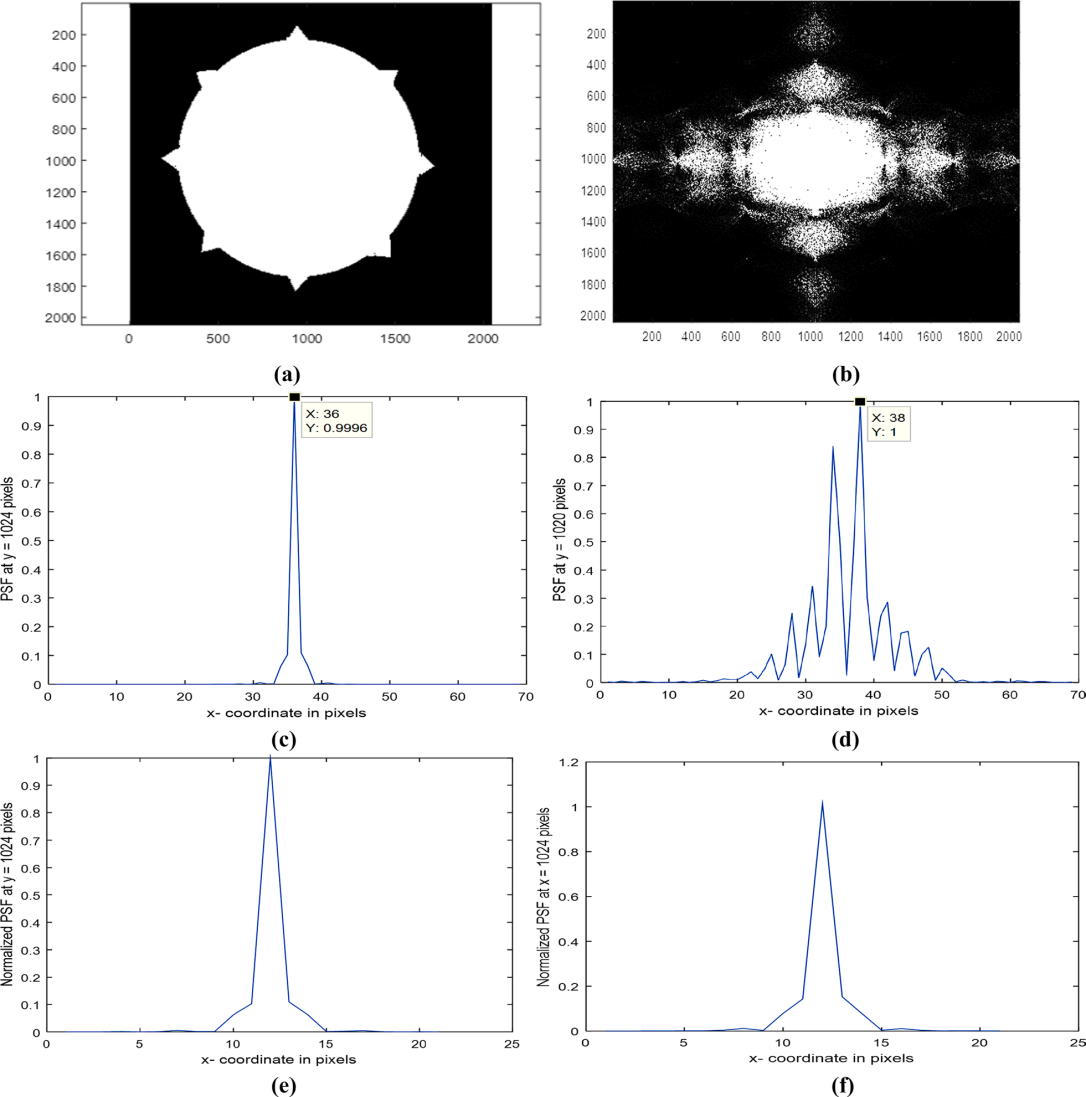
**a** Uniform circular aperture with 8 spikes in a matrix of dimensions 2048 × 2048 pixels. The average diameter = 1360 pixels. **b**: The diffracted image corresponding to the aperture is shown in (**a**). **c**: The Normalized PSF plot in the range from 990 up to 1058 pixels corresponding to the image is shown in the **a**. **d**: The Normalized PSF plot in the range from 990 up to 1058 pixels at y = 1020 pixels corresponding to the image is shown in **a**. **e**: The Normalized PSF plot in the range from 1014 up to 1034 pixels at y = 1024 pixels corresponding to the image is shown in **a**. **f**: The Normalized PSF plot in the range from 1014 up to 1034 pixels at *x* = 1024 pixels for the PSF image is shown in (**a**)

Another uniform circular aperture with 8 spikes in a matrix of dimensions 2048 × 2048 pixels while the average diameter = 1360 pixels is plotted as shown in Fig. 3a. The diffracted image corresponding to the aperture is plotted as shown in Fig. 3b.

The Normalized PSF plot in the range from 990 up to 1058 pixels corresponding to the image is shown in Fig. 3a and Fig. 3c. It is shown that the central peak is found at 36 pixels, while the normalized PSF plot in the range from 990 up to 1058 pixels corresponding to the image is shown in Fig. 3a. It is shown that the central peak is found at 38 pixels and hence shifted by 4 pixels around center as compared with Fig. 3c. In addition, different strong legs are shown. The shift attributed to the plot at 1020 pixels which are shifted from the center at 1024 pixels.

The Normalized PSF plot in the range from 1014 up to 1034 pixels at *y* = 1024 pixels corresponding to the image is shown in Fig. 3a plotted as in Fig. 3e, while the normalized PSF plot in the range from 1014 up to 1034 pixels at *x* = 1024 pixels is shown in Fig. 3f.

**Fig. 4:**
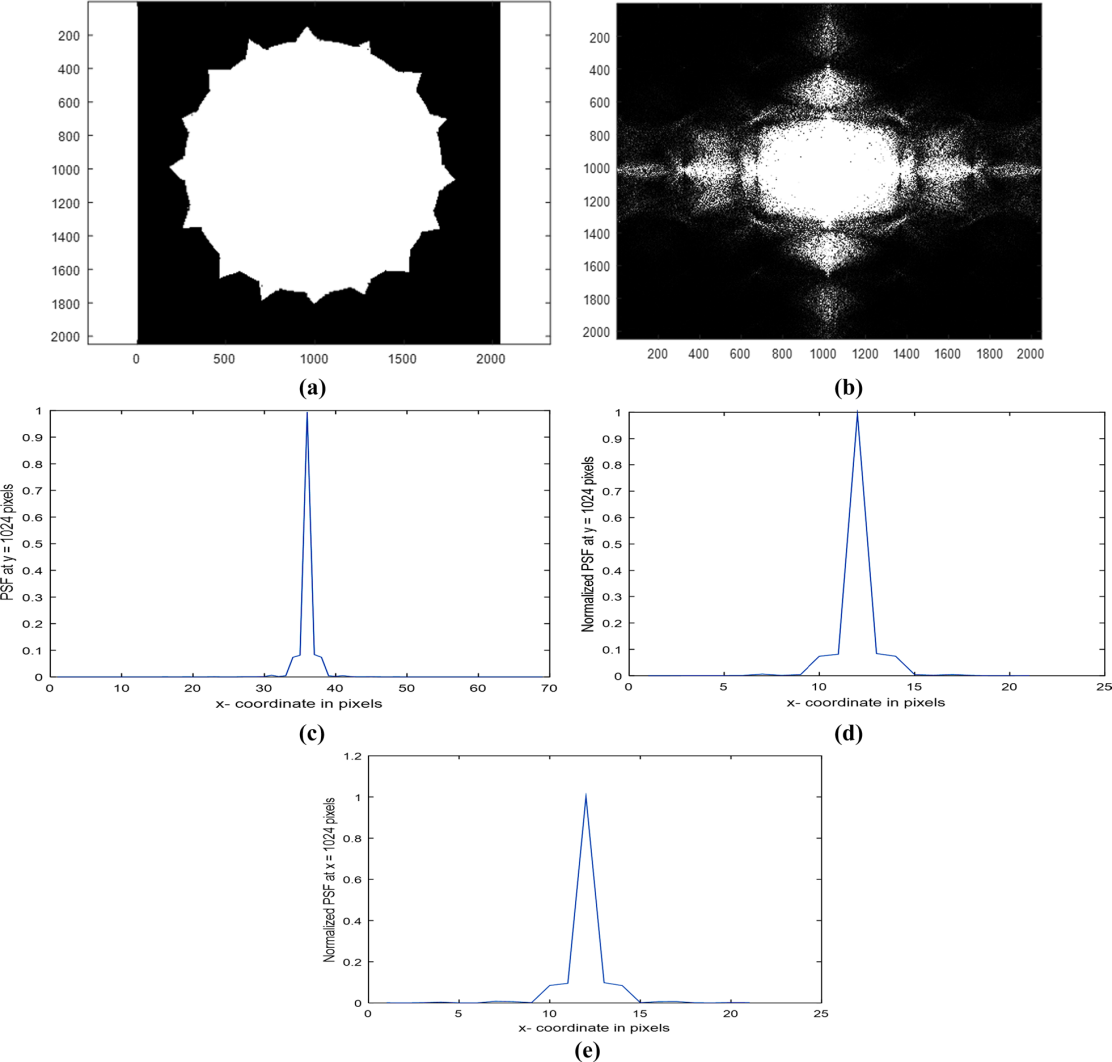
**a** Uniform circular aperture with 16 spikes in a matrix of dimensions 2048 × 2048 pixels. The average diameter = 1445 pixels. **b**: The diffracted image or the intensity impulse response is corresponding to the aperture of 16 spikes shown in (**a**). **c**: The Normalized PSF plot in the range from 990 up to 1058 pixels is corresponding to the image shown in the (4- a). (4- d): The Normalized PSF plot in the range from 1014 up to 1034 pixels at y = 1024 pixels is corresponding to the image show4n in (**a**). **e**: The Normalized PSF plot in the range from 1014 up to 1034 pixels at x = 1024 pixels is corresponding to the image shown in (**a**)

Similar plots are plotted for the PSF, using the circular aperture but with 16 spikes in a matrix of dimensions 2048 × 2048 pixels and average diameter = 1445 pixels. Figure 4a is plotted and Fig. 4b–e is plotted. The diffracted image or the intensity impulse response corresponding to the aperture of 16 spikes is shown in Fig. 4b. The Normalized PSF plot in the range from 990 up to 1058 pixels corresponding to the image is shown in Fig. 4a and Fig. 4c. It is shown that the central peak is found at 36 pixels, while the normalized PSF plot in the range from 1014 up to 1034 pixels at y = 1024 pixels is shown in Fig. 4d and at *x* = 1024 pixels shown in Fig. 4e.

**Fig. 5:**
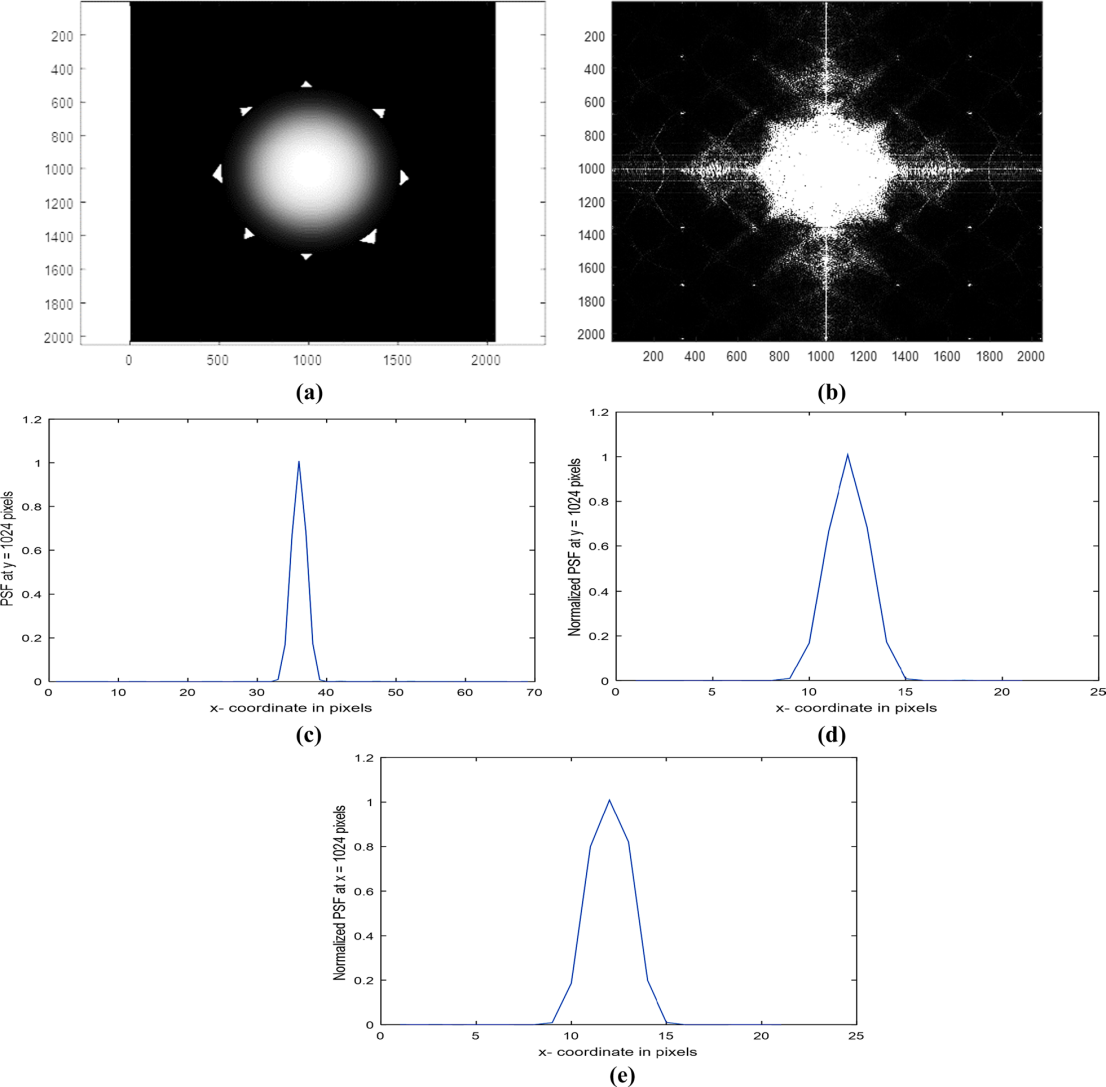
**a** Circular aperture of linear distribution with 8 spikes in a matrix of dimensions 2048 × 2048 pixels. The average diameter = 1024 pixels. **b**: The PSF image or intensity impulse response is corresponding to the aperture shown in (**a**). **c**: The Normalized PSF plot in the range from 990 up to 1058 pixels is corresponding to the image shown in (**a**). It is shown that the central peak is found at 36 pixels. **d**: The Normalized PSF plot in the range from 1014 up to 1034 pixels at *y* = 1024 pixels is corresponding to the image shown in (**a**). **e**: The Normalized PSF plot in the range from 1014 up to 1034 pixels at *x* = 1024 pixels is corresponding to the image shown in (**a**)

A circular aperture of linear distribution with 8 spikes in a matrix of dimensions 2048 × 2048 pixels is shown in Fig. 5a. Its diffracted image is shown in Fig. 5b. The Normalized PSF plot in the range from 990 up to 1058 pixels is corresponding to the image shown in Fig. 5a and Fig. 5c. It is shown that the central peak is found at 36 pixels, while the normalized PSF plot in the range from 1014 up to 1034 pixels at *y* = 1024 pixels is shown in Fig. 5d and at *x* = 1024 pixels shown in Fig. 5e.

**Fig. 6:**
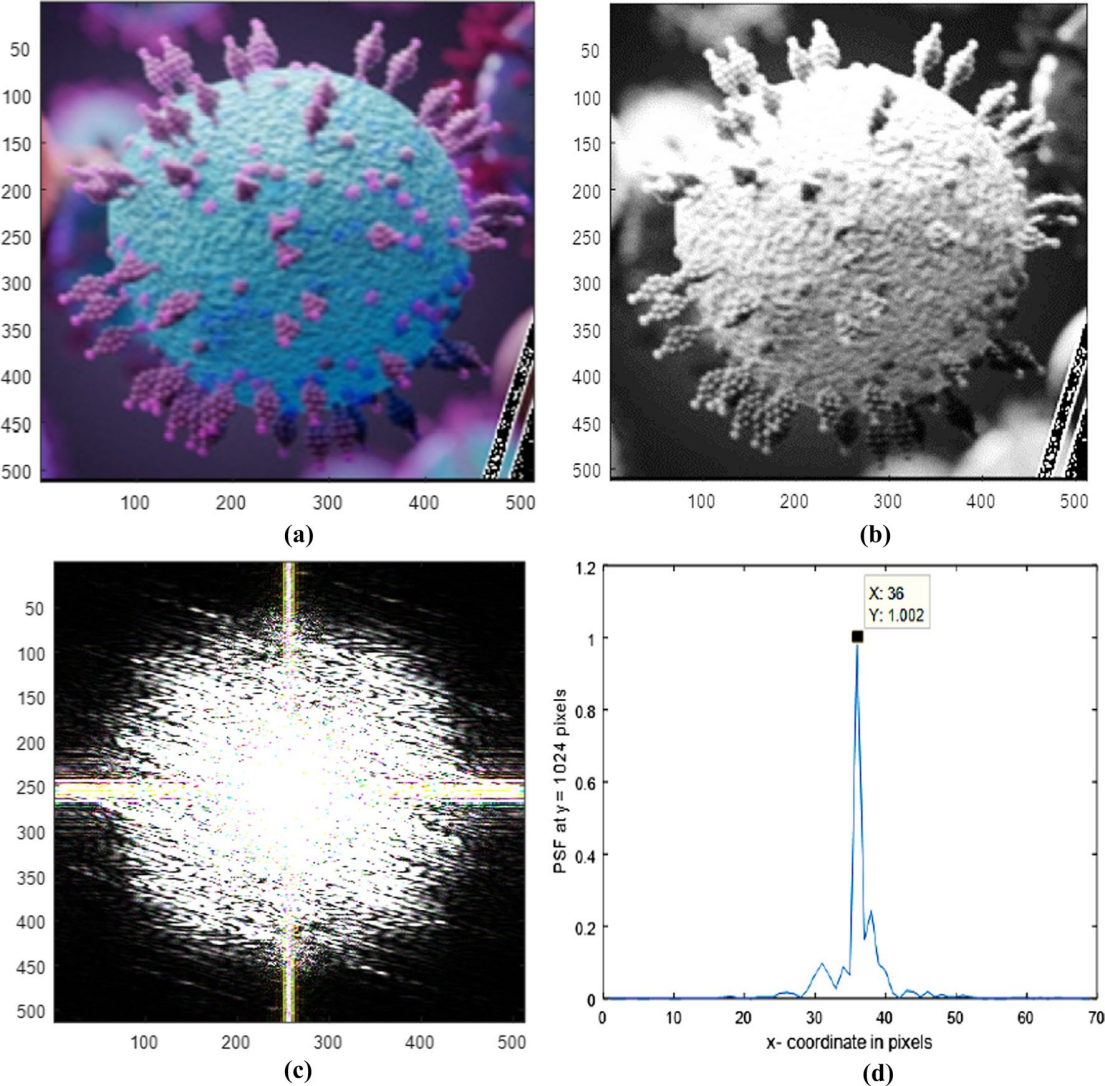
a Color image of omicron corona virus image with spikes in a matrix of dimensions 512 × 512 pixels. **b**: Gray scale image of omicron corona virus image with spikes in a matrix of dimensions 512 × 512 pixels. The average diameter = 340 pixels. **c**: The diffracted image corresponding to the omicron covid 19, shown in **b**, of matrix dimensions 512 × 512 pixels. **d**: The Normalized PSF plot in the range from 990 up to 1058 pixels corresponding to the Omicron corona virus image shown in (**a**)

Color image of omicron corona virus image with spikes in a matrix of dimensions 512 × 512 pixels is shown in Fig. 6a. Gray scale image of omicron corona virus image with spikes in a matrix of dimensions 512 × 512 pixels is shown in Fig. 6b. The average diameter = 340 pixels.

The corresponding diffracted image is shown in Fig. 6c, of matrix dimensions 512 × 512 pixels. The Normalized PSF plot in the range from 990 up to 1058 pixels is corresponding to the Omicron corona virus image shown in Fig. 6d.

**Fig. 7:**
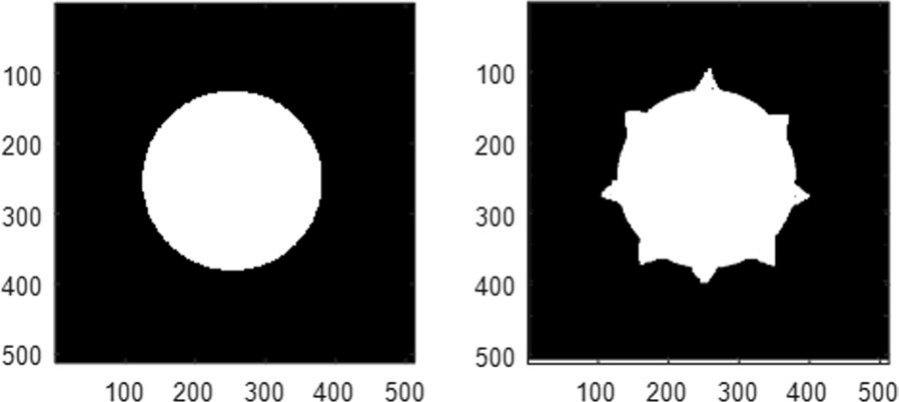
Two apertures corresponding to the confocal microscope lenses. In the L.H.S., circular aperture is shown while in the R.H.S., the aperture is provided with 8 spikes (1st model of Covid 19). The two apertures have the same sizes of radius = 128 pixels and the whole matrix has dimensions of 512 × 512 pixels

Two apertures corresponding to the confocal microscope lenses are shown in Fig. 7. In the L.H.S., circular aperture is shown while in the R.H.S., the aperture is provided with 8 spikes (1^st^ model of Covid 19). The two apertures have the same sizes of radius = 128 pixels, and the whole matrix has dimensions of 512 × 512 pixels.

**Fig. 8:**
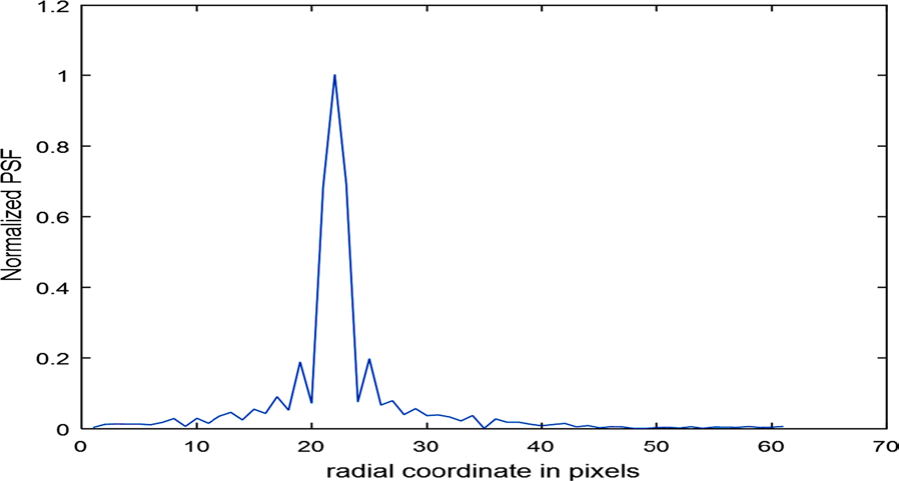
Normalized PSF for the spiky aperture shown in the R.H.S. of (7). The radial coordinate range is from 236 up to 296 pixels of total range 60 pixels where the center is at 256 pixels. Hence, in the figure the peak is found at 22 pixels which deviate from the theoretical value by 2 pixels

The normalized PSF, for the spiky aperture in the R.H.S. of Fig. 7, is shown in Fig. 8. The radial coordinate range is from 236 up to 296 pixels of total width = 60 pixels where the center is at 256 pixels.

**Fig. 9:**
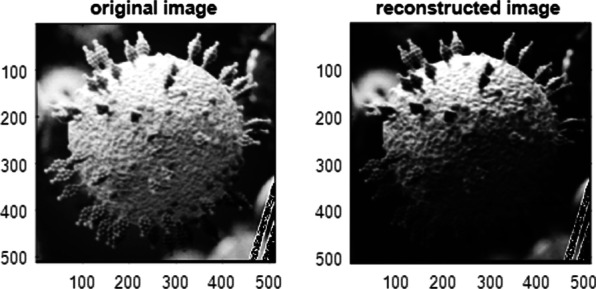
In the left, the original image of corona virus is shown while in the right, the reconstructed image is obtained using confocal laser microscope provided with the apertures shown in (7)

In Fig. 9, in the L.H.S. the original image of corona virus is shown while in the R.H.S., the reconstructed image is obtained using CSLM provided with the apertures shown in Fig. 7.

**Fig. 10:**
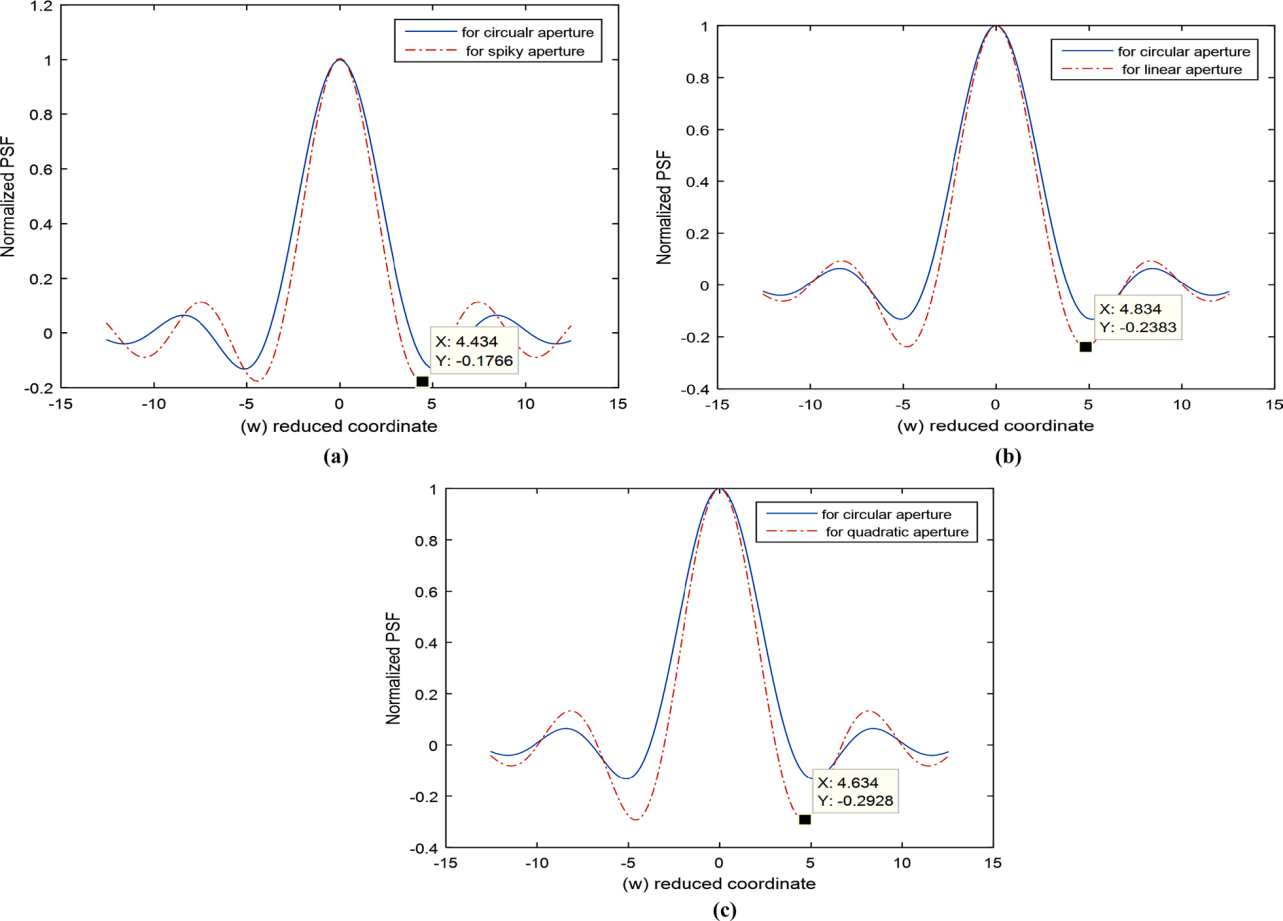
**a** Normalized PSF computed from Eq. (8) for the spiky pattern where *β* = 0.5 computed from Eq. (12) for the circular aperture. The same reduced coordinate range is taken constant from − 4*pi to 4*pi in both plots. **b**: The Normalized PSF for the linear aperture compared with that corresponding to the circular aperture. The same reduced coordinate range is taken constant from − 4*pi to 4*pi in both plots. **c**: The Normalized PSF for the quadratic aperture is compared with that corresponding to the circular aperture. The same reduced coordinate range is taken constant from − 4*pi to 4*pi in both plots

The normalized PSF is computed from Eq. (8) for the spiky pattern where *β* = 0.5 is computed from Eq. (12) for the circular aperture. The same reduced coordinate range taken constant from -4π to 4π in both plots is shown in Fig. 10a. It is shown in Fig. 10a that cutoff spatial frequency in reduced coordinate is computed as follows:

$$W_{c} = 4.434 ,{\text{for}} {\text{the spiky aperture and}} W_{c} = 5.234 {\text{for the circular aperture}}.$$

The PSF plots are shown in Figs. 10 b, c corresponding to linear and quadratic apertures [2, 3] for comparison with the plot in Fig. 10a corresponding to the spiky aperture. The PSF corresponding to the uniform circular aperture is shown in all plots for comparison.

## Discussion

The irregular surface of spiky shape for the circular aperture, shown in Fig. 2a, is investigated since it has resemblance with the corona virus. The diffracted image in Fig. 2b showed moderate intense legs surrounding the central intense peak. The normalized PSF shown in Fig. 2c has central peak of triangular shape surrounded by legs of irregular distribution and weak intensity compared with the central peak intensity. To see the irregularity outside the central peak, the plot in the range from 1040 up to 1100 pixels is shown in Fig. 2d at constant *x* = 1024 pixels and in Fig. 2e at constant *y* = 1024 pixels. The irregular distribution of the legs is attributed to the presence of irregular spikes surrounding the circular aperture. Hence, the PSF outside the central peak corresponding the model 1 Fig. 2a showed different irregular distributions as shown in Figs. 2d, e.

The diffracted image is computed and plotted as shown in Fig. 3b corresponding to the uniform circular aperture with 8 spikes shown in Fig. 3a. The diffracted image has intense central peak surrounded by weak intense legs. Figures shown in Fig. 3c–f represent plots for the normalized PSF under different conditions. The plot in the range from 990 up to 1058 pixels at *y* = 1024 pixels shown in Fig. 3c has central peak at 36 pixels of rectangular shape with inclined wider width of linear distribution, while no diffracted legs are shown in the plot. It is shown in Fig. 3d that the central peak is found at 38 pixels and hence shifted by 4 pixels around center as compared with Fig. 3c. In addition, different strong legs are shown. The shift is attributed to the plot taken at 1020 pixels which are shifted from the center at 1024 pixels. Hence, using the uniform circular aperture provided with 8 spikes, it is shown 4 peaks on both sides of the central peak. Referring to Fig. 3e, it is shown central triangular peak and wider curved shape near the edges reaching zero at the cutoff of the diffraction pattern. The plot is taken at *y* = const, while in Fig. 3f, it is shown central peak followed by wider linear shape near the edges reaching zero at the cutoff of the diffraction pattern. In addition, on peak of weak intensity on both sides it is shown where *x* = const.

The plots corresponding to the circular aperture with 16 spikes are shown in Fig. 4a and Fig. 4c–e. The diffracted image shown in Fig. 4b is brighter than that corresponding with only 8 spikes. It is shown that the central peak is found at 36 pixels in all plots including Fig. 4c in the range from 990 up to 1058 pixels. In addition, the central peak has triangular shape followed by a constant value and then drops linearly reaching at the cutoff from the diffraction pattern as shown in Fig. 4d, e in the interval from 1014 up to 1034 pixels. It is shown that the central peak surrounded by a linear decreased shape in case of 8-spiky aperture (Fig. 3c, e, f), while it is surrounded by a flat base followed by linear decreased shape in case of 16-spiky aperture, Fig. 4c, d, e. Equal cutoff shown in the PSF plots is taken at either *y* = 1024 pixels or *x* = 1024 pixels, respectively. Hence, the PSF plot shows difference as the number of spikes increases.

The diffracted image shown in Fig. 5b corresponding to the circular aperture of linear distribution provided with 8 spikes has central peak with spikes compared with the rectangular shapes shown in Figs. 3b, 4b. In addition, diffracted spots appear in Fig. 5b due to the presence of intense spikes in the aperture. The normalized PSF plot in the range from 990 up to 1058 pixels corresponding to the image is shown in Fig. 5a and Fig. 5c. It is shown that the central peak found at 36 pixels and has triangular shape, while the Normalized PSF plot, in the range from 1014 up to 1034 pixels at *y* = 1024 pixels, is shown in Fig. 5d and at *x* = 1024 pixels shown in Fig. 5e, having a near rocket shape.

The diffracted image shown in Fig. 6c corresponding to omicron corona virus image is shown in Fig. 6a, b The diffracted image has randomness in the central peak which is attributed to the vast number of spikes in the corona virus image. The Normalized PSF plot in the range from 990 up to 1058 pixels has central peak found at 36 pixels as shown in Fig. 6d. In addition, the legs randomly distributed of irregular asymmetric shape depend on the image shape.

The Normalized PSF, for the spiky aperture in the R.H.S. of Fig. 7, is shown in Fig. 8. The radial coordinate range is from 236 up to 296 pixels of total width = 60 pixels where the center is at 256 pixels. Hence, in the figure the peak is found at 22 pixels which deviates from the theoretical value by 2 pixels.

The reconstructed image of corona virus, using CSLM provided with one spiky aperture and the other of uniform circular, is shown in Fig. 9.

Referring to Fig. 10a, the Normalized PSF corresponding to the spiky aperture and the uniform circular aperture showed resolution improvement for the spiky aperture since the spiky zone approximated is by annular shape with amplitude decided by the parameter *β*.

The cutoff reduced coordinate $$W_{c} = 4.434 {\text{for spiky aperture compared with}} W_{c} = 5.134 {\text{for circular aperture}}$$$$.$$ Resolution improvement is attained in case of spiky aperture realized by computing the PSF from Eq. (8) and comparing it with the computed PSF for circular aperture represented by the Airy disc.

Referring to Fig. 10a, b and c) corresponding to the PSF, we get these cutoff values:

$$W_{c} \left( {{\text{circluar}}} \right) = 5.134, \;W_{c} \left( {{\text{linear}}} \right) = 4.834, \;W_{c} \left( {{\text{quadratic}}} \right) = 4.634, W_{c} \left( {{\text{spiky}}} \right) = 4.434.$$

Hence, it shown that the cutoff for the PSF corresponding to the spiky aperture is sharper than in case of uniform circular aperture and modulated linear and quadratic apertures.

## Conclusions

The proposal of irregular distribution around the uniform circular aperture and around the circular apertures with linear distribution has a great similarity with the corona virus images. Hence, a study of models of irregular spikes, and other models of definite number of spikes, 8 and 16 spikes, surrounding the apertures, is carried out. The resultant PSF for the microscope lenses, where the 1st has a spiky pattern while the 2nd has uniform circular distribution, is computed in the section of methods. The impulse response or the PSF corresponding to the apertures of definite spikes shows diffracted legs dependent on the number of spikes. In addition, the PSF plot shows difference as the number of spikes increases. It is shown that the central peak is surrounded by a linear decreased shape in case of 8-spiky aperture Fig. 3 c, e, f, while it is surrounded by a flat base followed by linear decreased shape in case of 16-spiky aperture, Fig. 4 c, d, e. There is equal cutoff shown in the PSF plots, taken at either *y *= 1024 pixels or *x* = 1024 pixels, respectively. A resolution improvement reached in case of spiky aperture is compared with circular aperture.

In addition, the impulse response corresponding to omicron corona virus image showed randomness in the central peak of the diffraction pattern and its PSF has central peak surrounded by irregular legs, Fig. 6d. We apply the spiky apertures in the formation of images in the confocal laser microscope. The aperture with 8 spikes is placed in front of the 1st microscope lens while the uniform circular aperture is placed behind the 2^nd^ lens facing the point detector. The mechanical scanning of the image (omicron corona virus) synchronized with the electronic scanning of the detector allows forming the image in the CSLM. It is proved that the spiky aperture used for laser illumination gives PSF better than the PSF for linear, quadratic, and circular apertures. This is shown from different cutoff values in the PSF corresponding to different apertures. Consequently, resolution improvement is reached in the reconstructed images with spiky aperture.

## Data Availability

I confirm that all relevant data are included in the article since the article describes entirely theoretical research.

## Abbreviations

F.T: Fourier transform
FFT: Fast Fourier transform
PSF: Point spread function
CSLM: Confocal scanning laser microscope

## References

[1] Sheppard CJR, Wilson T (1979). Imaging properties of annular lenses. Appl Opt.

[2] Hamed AM, Clair JJ (1983). Image, and super-resolution in optical coherent microscopes. Optik.

[3] Hamed AM, Clair JJ (1983). Studies on optical properties of confocal scanning optical microscope using pupils with radially transmission distribution. Optik.

[4] Hamed AM (2009). Numerical speckle images formed by diffusers using modulated conical and linear apertures. J Mod Opt.

[5] Hamed AM (2017). Improvement of point spread function (PSF) using linear quadratic aperture. Optik.

[6] Hamed AM (2019). Design of a Cascaded Black – Linear Distribution (CBLD) in Circular aperture and its application on confocal laser scanning microscope (CLSM). Am J Opti Photon.

[7] Hamed AM, Al-Saeed TA (2021). Reconstruction of images in non-scanned confocal microscope (NSCM) using speckle imaging. Beni-Suef Univ J Basic Appl Sci.

[8] Gu M, Sheppard CJR, Zhou H (1993). Optimization of axial resolution in confocal imaging using annular pupils. Optik.

[9] Biao Xu, Zhejiang Wang, and Jinping He. Super-resolution imaging via aperture modulation and intensity extrapolation. www.nature.com/scientific reports published on 18 October 2018 10.1038/s41598-018-33416-9 PMC6185970 30315198

[10] Nasrollahi K, Moeslund TB (2014). Super-resolution: a comprehensive survey. Mach Vis Appl.

[11] Mboula FMN, Starck JL, Ronayette S, Okumura K, Amiaux J (2015). Super-resolution method using sparse regularization for point-spread function recovery. Astron Astro Phys.

[12] Hirsch M, Harmeling S, Sra S, Scholkopf B (2011). Online multi-frame blind deconvolution with super-resolution and saturation correction. Astron Astro Phys.

[13] Puschmann KG, Kneer F (2005). On super-resolution in astronomical imaging. Astron Astro Phys.

[14] Wang C (2015). Super-resolution optical telescopes with local light diffraction shrinkage. Sci Rep.

[15] Djilali S (2020). Modeling the impact of unreported cases of the COVID-19 in the north African countries. Biology.

[16] Bentout S (2021). Age-structured modeling of COVID-19 epidemic in the USA, UAE, and Algeria. Alexandria Eng J.

[17] Zeb A (2021). Analysis of a discrete mathematical COVID-19 model. Res Phys.

[18] Zeb A (2022). A robust study of a piecewise fractional order COVID-19 mathematical model. Alexandria Eng J.

[19] Djilali S (2021). Approximating the asymptomatic infectious cases of the COVID-19 disease in Algeria and India using a mathematical model. Int J Model Simul nd Sci Comput.

[20] Comparing and contrasting the Omicron and Delta Plus variants of COVID-19 (carehospitals.com)

[21] Born M and Wolf E, Principles of optics, p.415 (1959)

